# 3-Eth­oxy-2-hy­droxy­benzaldehyde 2,4-di­nitro­phenylhydrazone *N*,*N*-di­methyl­formamide monosolvate

**DOI:** 10.1107/S1600536810029983

**Published:** 2010-08-04

**Authors:** Lin-xiu Zhao, Jian-lan Cui, Duan-lin Cao

**Affiliations:** a College of Chemical Engineering and Environment, North University of China, Taiyuan 030051, People’s Republic of China

## Abstract

The Schiff base of the title compound, C_15_H_14_N_4_O_6_·C_3_H_7_NO, was obtained from the condensation reaction of 3-eth­oxy-2-hy­droxy­benzaldehyde and 2,4-dinitro­phenyl­hydrazine. The dihedral angle between the benzene rings is 3.05 (10)° and intra­molecular N—H⋯O and O—H⋯O hydrogen bonds generate *S*(6) and *S*(5) ring motifs, respectively. In the crystal, the Schiff base and dimethyl­formamide solvent mol­ecules are linked by an O—H⋯O hydrogen bond.

## Related literature

For a related structure and background references, see: Zhao *et al.* (2010 ▶).
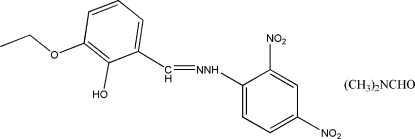


## Experimental

### 

#### Crystal data

C_15_H_14_N_4_O_6_·C_3_N_7_NO
*M* *_r_* = 419.40
Triclinic, 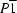

*a* = 7.1070 (6) Å
*b* = 7.7200 (7) Å
*c* = 19.4790 (19) Å
α = 84.677 (7)°
β = 81.562 (7)°
γ = 68.707 (8)°
*V* = 984.10 (16) Å^3^
*Z* = 2
Mo *K*α radiation
μ = 0.11 mm^−1^
*T* = 293 K
0.20 × 0.18 × 0.17 mm

#### Data collection

Bruker SMART CCD diffractometer
Absorption correction: multi-scan (*SADABS*; Bruker, 1998 ▶) *T*_min_ = 0.974, *T*_max_ = 0.978
6788 measured reflections
4011 independent reflections
1655 reflections with *I* > 2σ(*I*)
*R*_int_ = 0.028

#### Refinement

*R*[*F* ^2^ > 2σ(*F* ^2^)] = 0.039
*wR*(*F* ^2^) = 0.076
*S* = 0.74
4011 reflections
271 parameters
H-atom parameters constrained
Δρ_max_ = 0.12 e Å^−3^
Δρ_min_ = −0.23 e Å^−3^

### 

Data collection: *SMART* (Bruker, 1998 ▶); cell refinement: *SAINT* (Bruker, 1998 ▶); data reduction: *SAINT*; program(s) used to solve structure: *SHELXTL* (Sheldrick, 2008 ▶); program(s) used to refine structure: *SHELXTL*; molecular graphics: *SHELXTL*; software used to prepare material for publication: *SHELXTL*.

## Supplementary Material

Crystal structure: contains datablocks global, I. DOI: 10.1107/S1600536810029983/hb5578sup1.cif

Structure factors: contains datablocks I. DOI: 10.1107/S1600536810029983/hb5578Isup2.hkl

Additional supplementary materials:  crystallographic information; 3D view; checkCIF report

## Figures and Tables

**Table 1:** Hydrogen-bond geometry (Å, °)

*D*—H⋯*A*	*D*—H	H⋯*A*	*D*⋯*A*	*D*—H⋯*A*
N2—H2*A*⋯O3	0.86	2.01	2.6349 (19)	128
O1—H1*B*⋯O2	0.82	2.21	2.6581 (15)	115
O1—H1*B*⋯O7^i^	0.82	1.98	2.726 (2)	150

Symmetry code: (i) 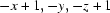.

**Fig. 1.**
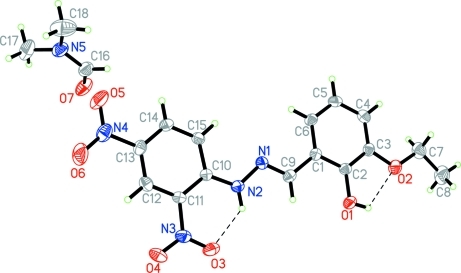
: the molecular structure of (I) with displacement ellipsoids drawn at the 30% probability level. Intramolecular hydrogen bonds are shown as dashed lines.

## supplementary crystallographic information

### Comment

As part of our ongoing studies of Schiff bases (Zhao *et al.*, 2010), we have synthesized the title compound, (I), and determined its crystal structure.

The molecular structure of (I) is shown in Fig.1. The benzene ring and the 2,4-dinitro benzene ring is nearly planar, making a dihedral angle of 3.05 (10)°.

Intramolecular N—H···O and O—H···O hydrogen bonds (Table 2) help to establish the planar conformation of the molecule.

### Experimental

2,4-Dinitrophenylhydrazine (1 mmol, 0.198 g) was dissolved in anhydrous ethanol (15 ml), H_2_SO_4_(98%, 0.5 ml) was then added and The mixture was stirred for several minitutes at 351k, 3-Ethoxy-2-hydroxybenzaldehyde (1 mmol, 0.166 g) in ethanol (8 mm l) was added dropwise and the mixture was stirred at refluxing temperature for 2 h. The product was isolated and recrystallized from DMF, red blocks of (I) were obtained after one week.

### Refinement

All H atoms were positioned geometrically and refined as riding with C—H = 0.93 (aromatic), 0.97 (methylene), 0.96 Å (methyl) and N—H = 0.86 Å, with *U*_iso_(H) = 1.2*U*_eq_(CH, CH2 or NH) and *U*_iso_(H) = 1.5*U*_eq_(C).

### Crystal data

**Table:**

C_15_H_14_N_4_O_6_·C_3_N_7_NO	*Z* = 2
*M**_r_* = 419.40	*F*(000) = 440
Triclinic, *P*1	*D*_x_ = 1.415 Mg m^−^^3^
Hall symbol: -P 1	Mo *K*α radiation, λ = 0.71073 Å
*a* = 7.1070 (6) Å	Cell parameters from 1739 reflections
*b* = 7.7200 (7) Å	θ = 3.1–26.4°
*c* = 19.4790 (19) Å	µ = 0.11 mm^−^^1^
α = 84.677 (7)°	*T* = 293 K
β = 81.562 (7)°	Block, red
γ = 68.707 (8)°	0.20 × 0.18 × 0.17 mm
*V* = 984.10 (16) Å^3^	

### Data collection

**Table:**

Bruker SMART CCD diffractometer	4011 independent reflections
Radiation source: fine-focus sealed tube	1655 reflections with *I* > 2σ(*I*)
graphite	*R*_int_ = 0.028
ω scans	θ_max_ = 26.4°, θ_min_ = 3.1°
Absorption correction: multi-scan (*SADABS*; Bruker, 1998)	*h* = −8→8
*T*_min_ = 0.974, *T*_max_ = 0.978	*k* = −9→9
6788 measured reflections	*l* = −14→24

### Refinement

**Table:**

Refinement on *F*^2^	Primary atom site location: structure-invariant direct methods
Least-squares matrix: full	Secondary atom site location: difference Fourier map
*R*[*F*^2^ > 2σ(*F*^2^)] = 0.039	Hydrogen site location: inferred from neighbouring sites
*wR*(*F*^2^) = 0.076	H-atom parameters constrained
*S* = 0.74	*w* = 1/[σ^2^(*F*_o_^2^) + (0.0323*P*)^2^] where *P* = (*F*_o_^2^ + 2*F*_c_^2^)/3
4011 reflections	(Δ/σ)_max_ < 0.001
271 parameters	Δρ_max_ = 0.12 e Å^−^^3^
0 restraints	Δρ_min_ = −0.23 e Å^−^^3^

### Special details

**Table:**

Geometry. All e.s.d.'s (except the e.s.d. in the dihedral angle between two l.s. planes) are estimated using the full covariance matrix. The cell e.s.d.'s are taken into account individually in the estimation of e.s.d.'s in distances, angles and torsion angles; correlations between e.s.d.'s in cell parameters are only used when they are defined by crystal symmetry. An approximate (isotropic) treatment of cell e.s.d.'s is used for estimating e.s.d.'s involving l.s. planes.
Refinement. Refinement of *F*^2^ against ALL reflections. The weighted *R*-factor *wR* and goodness of fit *S* are based on *F*^2^, conventional *R*-factors *R* are based on *F*, with *F* set to zero for negative *F*^2^. The threshold expression of *F*^2^ > σ(*F*^2^) is used only for calculating *R*-factors(gt) *etc*. and is not relevant to the choice of reflections for refinement. *R*-factors based on *F*^2^ are statistically about twice as large as those based on *F*, and *R*- factors based on ALL data will be even larger.

### Fractional atomic coordinates and isotropic or equivalent isotropic displacement parameters (Å^2^)

**Table:**

	*x*	*y*	*z*	*U*_iso_*/*U*_eq_	
N2	0.7004 (2)	0.1384 (2)	0.44923 (8)	0.0440 (4)	
H2A	0.6798	0.2386	0.4699	0.053*	
N1	0.7508 (2)	−0.02993 (19)	0.48584 (9)	0.0428 (4)	
O1	0.82321 (18)	−0.01826 (15)	0.68544 (7)	0.0526 (4)	
H1B	0.8425	−0.0301	0.7264	0.079*	
O2	0.8899 (2)	−0.31145 (17)	0.77409 (7)	0.0581 (4)	
N3	0.6101 (2)	0.4898 (2)	0.36575 (11)	0.0510 (5)	
C1	0.8135 (3)	−0.1943 (2)	0.59413 (10)	0.0375 (5)	
C10	0.6832 (3)	0.1462 (2)	0.38088 (10)	0.0350 (5)	
C15	0.7047 (3)	−0.0166 (2)	0.34804 (10)	0.0412 (5)	
H15A	0.7291	−0.1278	0.3743	0.049*	
O3	0.6142 (2)	0.49976 (17)	0.42849 (8)	0.0680 (5)	
C3	0.8759 (3)	−0.3431 (3)	0.70742 (11)	0.0429 (5)	
C13	0.6554 (3)	0.1492 (3)	0.23938 (10)	0.0442 (5)	
O4	0.5797 (2)	0.62599 (17)	0.32584 (8)	0.0748 (5)	
C6	0.8332 (2)	−0.3652 (2)	0.56975 (10)	0.0435 (5)	
H6A	0.8181	−0.3735	0.5237	0.052*	
C11	0.6432 (3)	0.3112 (2)	0.33864 (11)	0.0380 (5)	
C12	0.6307 (3)	0.3106 (3)	0.26862 (11)	0.0455 (5)	
H12A	0.6054	0.4204	0.2415	0.055*	
C2	0.8389 (3)	−0.1847 (2)	0.66297 (11)	0.0390 (5)	
C9	0.7639 (2)	−0.0246 (2)	0.54994 (11)	0.0428 (5)	
H9A	0.7416	0.0885	0.5688	0.051*	
O5	0.6626 (2)	0.0037 (2)	0.14012 (8)	0.0852 (5)	
C14	0.6907 (3)	−0.0156 (2)	0.27938 (10)	0.0448 (5)	
H14A	0.7046	−0.1247	0.2591	0.054*	
O7	0.1362 (3)	−0.0784 (2)	0.18791 (8)	0.0778 (5)	
N4	0.6425 (3)	0.1503 (3)	0.16536 (10)	0.0631 (5)	
C4	0.8958 (3)	−0.5105 (3)	0.68260 (11)	0.0491 (6)	
H4A	0.9233	−0.6165	0.7120	0.059*	
C5	0.8748 (3)	−0.5210 (3)	0.61339 (11)	0.0494 (5)	
H5A	0.8890	−0.6345	0.5966	0.059*	
N5	0.3469 (3)	−0.1964 (2)	0.09163 (11)	0.0652 (5)	
O6	0.6127 (3)	0.2979 (2)	0.13149 (8)	0.0938 (6)	
C16	0.3049 (4)	−0.1592 (3)	0.15849 (13)	0.0624 (6)	
H16A	0.4130	−0.1980	0.1849	0.075*	
C8	0.9158 (4)	−0.3863 (3)	0.89285 (12)	0.0870 (8)	
H8A	0.9375	−0.4849	0.9280	0.131*	
H8B	0.7849	−0.2919	0.9041	0.131*	
H8C	1.0198	−0.3333	0.8905	0.131*	
C7	0.9238 (3)	−0.4618 (3)	0.82428 (11)	0.0698 (7)	
H7A	1.0557	−0.5570	0.8122	0.084*	
H7B	0.8201	−0.5167	0.8261	0.084*	
C18	0.5516 (4)	−0.2863 (3)	0.05946 (14)	0.1133 (10)	
H18A	0.6435	−0.3192	0.0941	0.170*	
H18B	0.5874	−0.2030	0.0248	0.170*	
H18C	0.5606	−0.3966	0.0381	0.170*	
C17	0.1878 (4)	−0.1365 (4)	0.04773 (13)	0.1068 (9)	
H17A	0.0594	−0.0790	0.0751	0.160*	
H17B	0.1852	−0.2420	0.0259	0.160*	
H17C	0.2122	−0.0485	0.0127	0.160*	

### Atomic displacement parameters (Å^2^)

**Table:**

	*U*^11^	*U*^22^	*U*^33^	*U*^12^	*U*^13^	*U*^23^
N2	0.0558 (12)	0.0373 (9)	0.0405 (12)	−0.0173 (8)	−0.0075 (9)	−0.0046 (8)
N1	0.0468 (11)	0.0406 (10)	0.0394 (11)	−0.0138 (8)	−0.0066 (9)	0.0001 (9)
O1	0.0710 (10)	0.0503 (8)	0.0399 (9)	−0.0223 (7)	−0.0123 (8)	−0.0057 (7)
O2	0.0787 (11)	0.0601 (9)	0.0364 (10)	−0.0255 (7)	−0.0109 (8)	0.0032 (8)
N3	0.0521 (12)	0.0397 (10)	0.0646 (15)	−0.0199 (8)	−0.0064 (11)	−0.0046 (11)
C1	0.0310 (12)	0.0441 (12)	0.0357 (13)	−0.0121 (9)	−0.0008 (10)	−0.0038 (10)
C10	0.0318 (12)	0.0361 (11)	0.0369 (13)	−0.0127 (9)	−0.0012 (10)	−0.0031 (10)
C15	0.0444 (13)	0.0343 (11)	0.0435 (14)	−0.0127 (9)	−0.0045 (11)	−0.0017 (10)
O3	0.0994 (13)	0.0524 (8)	0.0593 (12)	−0.0308 (8)	−0.0135 (10)	−0.0145 (8)
C3	0.0377 (13)	0.0500 (12)	0.0391 (14)	−0.0127 (10)	−0.0051 (11)	−0.0033 (12)
C13	0.0430 (13)	0.0568 (13)	0.0356 (14)	−0.0222 (10)	−0.0019 (11)	−0.0014 (12)
O4	0.1047 (12)	0.0379 (8)	0.0859 (13)	−0.0298 (8)	−0.0211 (10)	0.0116 (9)
C6	0.0410 (13)	0.0485 (11)	0.0371 (13)	−0.0104 (9)	0.0009 (10)	−0.0142 (11)
C11	0.0361 (12)	0.0322 (10)	0.0451 (14)	−0.0125 (9)	−0.0001 (11)	−0.0042 (10)
C12	0.0416 (13)	0.0453 (12)	0.0495 (15)	−0.0186 (10)	−0.0032 (11)	0.0081 (12)
C2	0.0339 (12)	0.0384 (11)	0.0436 (14)	−0.0112 (9)	−0.0023 (10)	−0.0076 (11)
C9	0.0412 (13)	0.0473 (12)	0.0415 (14)	−0.0173 (10)	−0.0017 (11)	−0.0086 (11)
O5	0.1203 (15)	0.1035 (12)	0.0488 (11)	−0.0576 (11)	−0.0052 (10)	−0.0211 (10)
C14	0.0449 (13)	0.0449 (12)	0.0431 (14)	−0.0143 (9)	0.0006 (11)	−0.0114 (11)
O7	0.0783 (12)	0.1091 (13)	0.0495 (11)	−0.0368 (10)	0.0068 (10)	−0.0288 (10)
N4	0.0707 (14)	0.0849 (14)	0.0423 (13)	−0.0399 (12)	−0.0008 (10)	−0.0045 (12)
C4	0.0444 (14)	0.0444 (12)	0.0520 (16)	−0.0096 (10)	−0.0038 (12)	0.0008 (11)
C5	0.0497 (14)	0.0396 (11)	0.0552 (16)	−0.0109 (9)	−0.0013 (12)	−0.0120 (11)
N5	0.0778 (16)	0.0675 (12)	0.0480 (14)	−0.0255 (11)	0.0068 (12)	−0.0146 (11)
O6	0.1387 (16)	0.1069 (13)	0.0483 (12)	−0.0604 (11)	−0.0215 (11)	0.0212 (10)
C16	0.079 (2)	0.0626 (15)	0.0550 (18)	−0.0345 (14)	−0.0119 (15)	−0.0054 (13)
C8	0.106 (2)	0.1054 (19)	0.0458 (18)	−0.0343 (16)	−0.0143 (16)	0.0096 (16)
C7	0.0826 (18)	0.0732 (15)	0.0496 (16)	−0.0253 (12)	−0.0120 (14)	0.0128 (14)
C18	0.104 (2)	0.097 (2)	0.125 (3)	−0.0310 (18)	0.041 (2)	−0.0406 (19)
C17	0.135 (3)	0.130 (2)	0.0534 (19)	−0.0400 (19)	−0.0210 (18)	−0.0117 (16)

### Geometric parameters (Å, °)

**Table:**

N2—C10	1.348 (2)	C12—H12A	0.9300
N2—N1	1.3751 (17)	C9—H9A	0.9300
N2—H2A	0.8600	O5—N4	1.228 (2)
N1—C9	1.271 (2)	C14—H14A	0.9300
O1—C2	1.3569 (19)	O7—C16	1.215 (2)
O1—H1B	0.8200	N4—O6	1.2263 (18)
O2—C3	1.368 (2)	C4—C5	1.391 (3)
O2—C7	1.4200 (19)	C4—H4A	0.9300
N3—O4	1.2204 (16)	C5—H5A	0.9300
N3—O3	1.2369 (19)	N5—C16	1.327 (3)
N3—C11	1.448 (2)	N5—C17	1.433 (3)
C1—C2	1.391 (2)	N5—C18	1.439 (3)
C1—C6	1.395 (2)	C16—H16A	0.9300
C1—C9	1.458 (2)	C8—C7	1.492 (3)
C10—C11	1.411 (2)	C8—H8A	0.9600
C10—C15	1.412 (2)	C8—H8B	0.9600
C15—C14	1.355 (2)	C8—H8C	0.9600
C15—H15A	0.9300	C7—H7A	0.9700
C3—C4	1.374 (2)	C7—H7B	0.9700
C3—C2	1.397 (2)	C18—H18A	0.9600
C13—C12	1.361 (2)	C18—H18B	0.9600
C13—C14	1.389 (2)	C18—H18C	0.9600
C13—N4	1.457 (2)	C17—H17A	0.9600
C6—C5	1.372 (2)	C17—H17B	0.9600
C6—H6A	0.9300	C17—H17C	0.9600
C11—C12	1.380 (3)		
			
C10—N2—N1	119.90 (16)	C13—C14—H14A	120.2
C10—N2—H2A	120.1	O6—N4—O5	123.4 (2)
N1—N2—H2A	120.1	O6—N4—C13	118.2 (2)
C9—N1—N2	115.76 (16)	O5—N4—C13	118.40 (19)
C2—O1—H1B	109.5	C3—C4—C5	119.78 (18)
C3—O2—C7	118.68 (16)	C3—C4—H4A	120.1
O4—N3—O3	121.94 (17)	C5—C4—H4A	120.1
O4—N3—C11	118.91 (18)	C6—C5—C4	120.52 (19)
O3—N3—C11	119.16 (16)	C6—C5—H5A	119.7
C2—C1—C6	119.10 (17)	C4—C5—H5A	119.7
C2—C1—C9	118.88 (18)	C16—N5—C17	120.2 (2)
C6—C1—C9	122.02 (19)	C16—N5—C18	122.2 (2)
N2—C10—C11	123.56 (18)	C17—N5—C18	117.5 (2)
N2—C10—C15	119.93 (16)	O7—C16—N5	125.1 (2)
C11—C10—C15	116.50 (19)	O7—C16—H16A	117.4
C14—C15—C10	121.93 (17)	N5—C16—H16A	117.4
C14—C15—H15A	119.0	C7—C8—H8A	109.5
C10—C15—H15A	119.0	C7—C8—H8B	109.5
O2—C3—C4	126.23 (18)	H8A—C8—H8B	109.5
O2—C3—C2	113.64 (18)	C7—C8—H8C	109.5
C4—C3—C2	120.1 (2)	H8A—C8—H8C	109.5
C12—C13—C14	120.79 (19)	H8B—C8—H8C	109.5
C12—C13—N4	119.36 (18)	O2—C7—C8	107.53 (18)
C14—C13—N4	119.8 (2)	O2—C7—H7A	110.2
C5—C6—C1	120.36 (19)	C8—C7—H7A	110.2
C5—C6—H6A	119.8	O2—C7—H7B	110.2
C1—C6—H6A	119.8	C8—C7—H7B	110.2
C12—C11—C10	121.16 (19)	H7A—C7—H7B	108.5
C12—C11—N3	116.24 (17)	N5—C18—H18A	109.5
C10—C11—N3	122.59 (19)	N5—C18—H18B	109.5
C13—C12—C11	119.91 (18)	H18A—C18—H18B	109.5
C13—C12—H12A	120.0	N5—C18—H18C	109.5
C11—C12—H12A	120.0	H18A—C18—H18C	109.5
O1—C2—C1	118.32 (16)	H18B—C18—H18C	109.5
O1—C2—C3	121.61 (19)	N5—C17—H17A	109.5
C1—C2—C3	120.06 (18)	N5—C17—H17B	109.5
N1—C9—C1	120.57 (19)	H17A—C17—H17B	109.5
N1—C9—H9A	119.7	N5—C17—H17C	109.5
C1—C9—H9A	119.7	H17A—C17—H17C	109.5
C15—C14—C13	119.69 (19)	H17B—C17—H17C	109.5
C15—C14—H14A	120.2		

### Hydrogen-bond geometry (Å, °)

**Table:**

*D*—H···*A*	*D*—H	H···*A*	*D*···*A*	*D*—H···*A*
N2—H2A···O3	0.86	2.01	2.6349 (19)	128
O1—H1B···O2	0.82	2.21	2.6581 (15)	115
O1—H1B···O7^i^	0.82	1.98	2.726 (2)	150

Symmetry codes: (i) −*x*+1, −*y*, −*z*+1.
